# Trace elements and lipidomic datasets of stranding samples in the blubber of Turpsios truncatus from the Yucatan coast: Association with biological features

**DOI:** 10.1016/j.dib.2023.109374

**Published:** 2023-07-06

**Authors:** Ixchel M. Ruiz-Hernández, Mohammad-Zaman Nouri, Marianne Kozuch, Nancy D. Denslow, Raúl E. Díaz-Gamboa, Rossanna Rodríguez-Canul, Reyna C. Collí-Dulá

**Affiliations:** a Departamento de Recursos del Mar, Centro de Investigación y de Estudios Avanzados del Instituto Politécnico Nacional, Unidad Mérida, Antigua Carretera a Progreso km 6, Cordemex, Mérida, Yucatán 97310, México; b Department of Physiological Sciences and Center for Environmental and Human Toxicology, University of Florida, PO Box 110885, 2187 Mowry Road. Gainesville, FL 32611, USA; c Departamento de Biología Marina, Universidad Autónoma de Yucatán, Mérida, Yucatán 97000, México; d Consejo Nacional de Ciencia y Tecnología (CONACYT), México

**Keywords:** Lipidomics, trace elements, bottlenose dolphin, blubber, ICP-MS

## Abstract

The Yucatan coastal zone is an area that contributes to many anthropogenic activities resulting in substantial contamination (metals, pesticides) in aquatic organisms. The dolphin is an excellent sentinel animal used in studying contamination in this area. Some substances found in dolphins have been identified as toxic causing alterations in the properties of membranes and produce lipid peroxidation especially heavy metals. The dataset presented here is associated with the research article paper entitled “Trace element and lipidomic analysis of bottlenose dolphin blubber from the Yucatan coast: Lipid composition relationships”. In this article, we presented the trace element concentrations found in blubber and their comparison with other studies performed in mammal marine organisms. Lipidomic characterization of bottlenose dolphin blubber and their association with trace elements and the differences related to biological characteristics were presented. This data provides a correlation analysis between trace element concentrations, lipid species and body length and the lipid differences related to biological characteristics such as growth stage, stranding code, and the presence of stomach contents. We used Spearman correlation analysis to identify the association with body length, trace elements and lipids. Wilcoxon rank-sum test was used to determine differences in lipids related to stranding code (3: moderate decomposition, 4: advanced decomposition), growth stage (juveniles and adults) and whether they showed presence of stomach contents or not. The data indicates that Cr, Cd and Zn concentrations were higher compared to concentrations found in blubbler of *T. truncatus* from other studies (See Table 3). Cr, Co, As and Cd were found in higher concentration in larger organisms compared to smaller ones. The results of correlation between lipids and body length showed a decrease in some ceramides (CER, DCER, HCER), sterols (CE), glycerolipids (TAG, DAG) and phosphatidylethanolamines (LPE, PE) in larger dolphins (Table 4). Dolphins with advanced decomposition (code 4) showed lower concentrations of phosphatidylethanolamines (PE) compared with organisms with moderate decomposition (code 3). Organisms with empty stomachs showed higher concentrations of phosphoethanolamines suggesting a preferential metabolism of energy-rich lipids over structural lipids. The information in these datasets may contribute to understanding the potential associations of trace elements, lipids and their associations with biological characteristics.

**Specifications Table**

**Table:** 

Subject area	Biology
More specific subject area	Trace elements Lipidomics
Type of data	Table, text file and figures.
How data was acquired	Trace elements data from inductively coupled plasma mass spectrometer (ICP-MS) and R software were used to identify correlations between trace elements and body length. Lipids data from LC-MS/MS chromatography, MultiQuant and Metaboanalyst and R softwares was used to identify differences between biological characteristics.
Data format	Filtered and analyzed.
Data source location	Sample sources were obtained from stranded dolphins between during 2016-2019 in the Yucatan coast in Gulf of Mexico. Lipid and trace elements extraction was carried out at University of Florida (Gainesville, FL., USA). Analysis was performed at University of Florida and CINVESTAV-Mérida, Yucatán, México.
Data accessibility	Lipidomics raw data are presented in MetaboLigths: https://www.ebi.ac.uk/metabolights/editor/MTBLS5772/descriptors
Related research article	Ruiz-Hernández, I. M., Nouri, M. Z., Kozuch, M., Denslow, N. D., Díaz-Gamboa, R. E., Rodríguez-Canul, R., & Collí-Dulá, R. C. (2022). Trace element and lipidomic analysis of bottlenose dolphin blubber from the Yucatan coast: Lipid composition relationships. Chemosphere, 299, 134353. https://doi.org/10.1016/j.chemosphere.2022.134353

## Value of the Data

• The data provides information of trace element concentrations found in blubber of bottlenose dolphin.
• Concentrations of Cr, Cd and Zn were higher in this study compared to concentrations in blubber of *T. truncatus* from other studies.
• Concentrations of Cr, Co, As and Cd, some ceramides, sterols, glycerolipids and phosphatidylethanolamines are influenced by the body length.
• The data explores the biological characteristics such as growth stage, stranding code, and presence of stomach contents, body length and their association with lipid concentrations.
• The data shown here is useful to other researchers that may explore the information about lipid differences between juveniles and adults, dolphins in moderate decomposition and advanced decomposition state, carcasses with stomach content and empty digestive tract.

## 1 Data Description

**Fig. 1:**
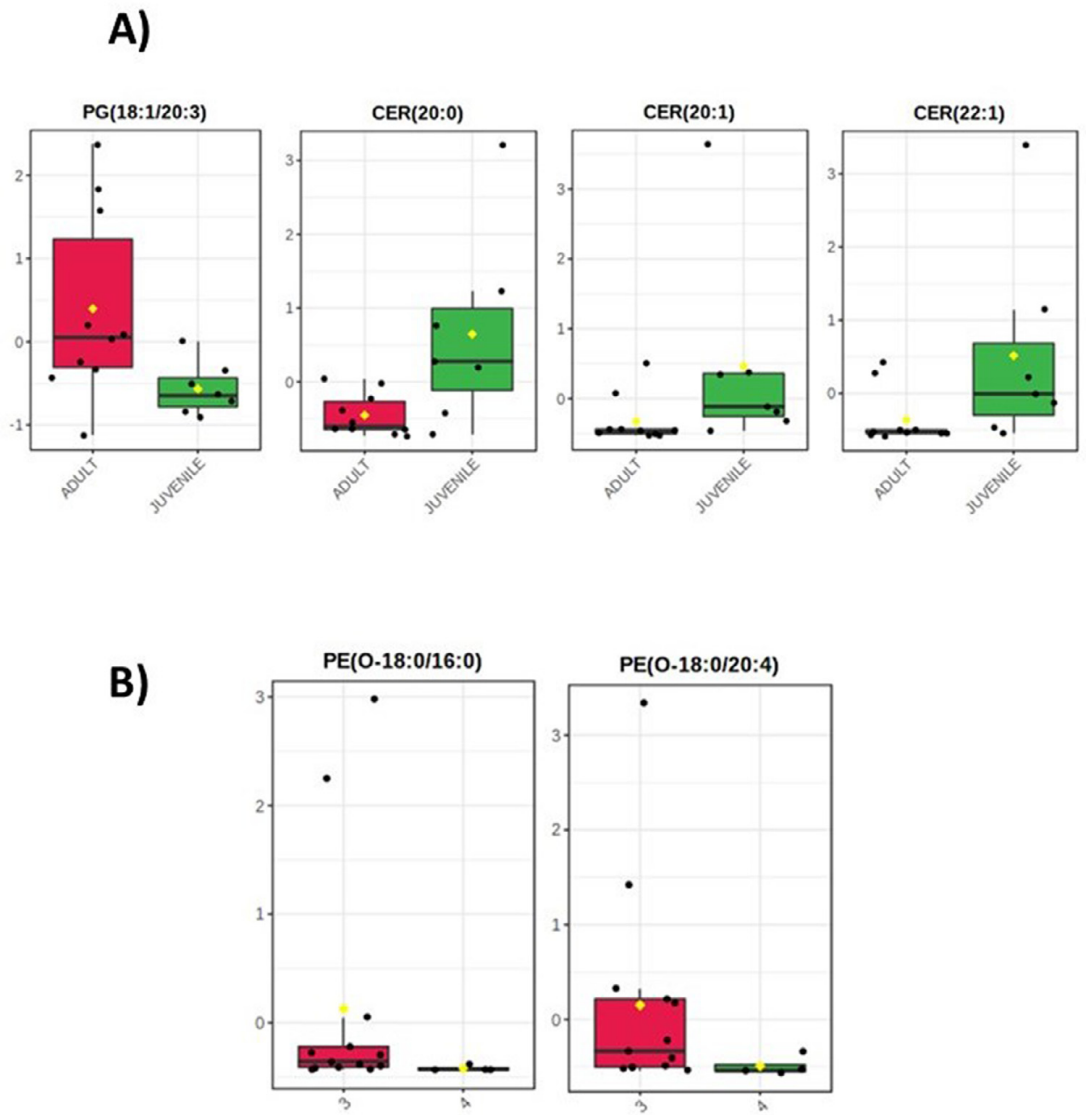
Lipid differences in relative concentrations (Wilcoxon rank-sum test) in blubber from the lateral region. (A) Differences between adults and juveniles. (B) Differences between stranding code 3 (moderate decomposition) and 4 (advanced decomposition).

**Fig. 2:**
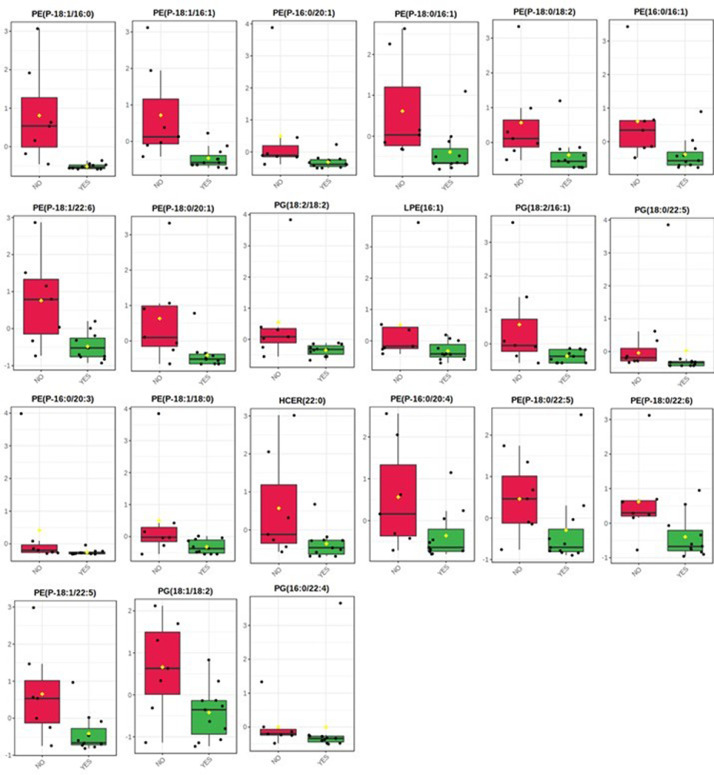
Lipid differences in relative concentrations (Wilcoxon rank-sum test) between dolphins with stomach content and without it.

These data sets provide information of the association between trace elements, lipids and biological characteristics in blubber from bottlenose dolphin. Table 1 shows biological attributes such as gender and body length in addition to the stranding information. Table 2 presents all the trace element concentrations found in each organism from both areas, lateral region and dorsal fin. Table 3 presents a comparison of the results from this study and the concentrations found in other studies. This data shows that Cr, Zn and Cd are higher in this study. Table 4 shows the significant correlations found between trace elements, lipids and body length. All significant differences between lipids and growth stage, stranding code, and stomach content are presented in Table 5, Fig. 1, Fig. 2.

**Table 1:** Biological data of bottlenose dolphin (*Tursiops truncatus*) stranded between 2016 and 2019 along the Yucatan coast.

Organism code	Stranding date	Gender	Total body length (cm)	Data source location	Stranding code
Tt8	05/02/2016	M	243	PROGRESO	2
Tt2	06/02/2016	F	280	SISAL	3
Tt1	07/02/2016	M	251	SISAL	3
Tt13	14/02/2016	M	242	PROGRESO	3
Tt3⁎	21/02/2016	UNKNOWN	234	PROGRESO	4
Tt6⁎	01/06/2016	F	120	CHUBURNÁ	3
Tt4	25/06/2016	F	249	DZILAM	4
Tt12⁎	11/10/2016	M	159	CHELEM	3
Tt10⁎	11/02/2017	M	278	CHUBURNÁ	3
Tt9	23/06/2017	M	108	PROGRESO	4
Tt7	12/07/2017	M	168	PROGRESO	3
Tt15	12/09/2017	F	260	SANTA CLARA	3
Tt14	27/09/2017	M	215	PROGRESO	3
Tt16	29/01/2018	F	242	CHELEM	3
Tt18	17/04/2018	F	213	CHUBURNÁ	3
Tt17	07/05/2018	M	189	CHELEM-CHUBURNÁ	3
Tt19	16/05/2018	F	245	CELESTÚN	3
Tt22	30/05/2018	F	201	CELESTÚN	3
Tt21	15/07/2018	M	265	CHICXULUB-UAYMITÚN	4
Tt23	24/07/2018	M	219	PROGRESO	4
Tt20	15/11/2018	M	240	CELESTÚN	3
Tt25	29/04/2019	F	183	SISAL	3

Stranding code: Code 1 (alive), Code 2 (fresh dead), Code 3 (moderately decomposed), Code 4 (advanced decomposition).

⁎ The lowest region of dorsal fin was collected only in these dolphins.

**Table 2:** Trace element concentrations (µg/g dry weight) mean, SD and range of each element, in blubber of bottlenose dolphins from the Yucatan coast, sampled from 2016 to 2019.

Organism	Cr	Mn	Fe	Co	Cu	Zn	As	Se	Cd
Concentrations in the lateral region below the dorsal fin									

Tt8	4.09	0.31	25.42	0.37	0.82	20.59	2.59	1.25	0.09
Tt2	4.07	0.76	25.99	0.11	0.72	9.2	2.97	1.02	0.11
Tt1	4.06	0.49	45.53	0.04	1.27	20.67	1.45	1.31	0.07
Tt13	6.4	0.45	52.63	0.05	4.26	57.21	1.99	1.47	0.04
Tt4	10.81	0.96	60.26	0.09	3.62	19.18	0.75	0.75	0.08
Tt9	2.89	0.85	80.4	0.02	3.29	150.58	0.93	1.21	ND
Tt7	1.79	0.34	18.99	0.01	1.07	21.03	0.55	1.21	ND
Tt15	8.8	1.07	117.94	0.08	1.61	26.54	1.49	2.56	0.17
Tt14	15.42	1.57	104.47	0.13	1.38	53.99	0.63	1.49	ND
Tt16	3.23	0.33	43.25	0.02	0.89	26.96	1.28	1.69	0.01
Tt18	2.04	0.28	25.17	0.01	0.75	12.11	1.83	2.65	ND
Tt17	2.37	0.38	19.85	0.02	0.72	18.29	0.66	0.5	ND
Tt19	10.18	1.32	86.65	0.12	1.45	55.6	0.59	1.33	0.02
Tt22	2.61	0.71	91.43	0.03	0.99	54.47	0.55	1.12	0.03
Tt21	4.17	0.64	52	0.04	1.46	57.6	1.67	1.9	0.12
Tt23	7.07	0.8	44.64	0.05	1.99	55.55	0.67	1.77	0.13
Tt20	3.95	0.49	65.05	0.03	2.31	31.84	0.73	2.6	0.13
Tt25	1.57	0.42	27.12	0.01	0.77	27.3	0.43	0.92	ND

Concentrations in dorsal fin									

Tt3	14.33	1.63	71.26	0.11	2	19.35	0.44	0.62	0.11
Tt6	11.79	1.14	75.49	0.09	2.41	39.64	0.34	0.86	0.08
Tt12	34.27	3.73	210.55	0.25	2.15	41.66	0.34	3.83	ND
Tt10	29.46	2.03	124.12	0.27	2.2	24.86	0.68	1.91	0.25

**Table 3:** Trace element concentrations (mean ± SD; ranges in parenthesis; µg/g dry weight, *wet weight) in blubber of bottlenose dolphin (*T. truncatus*) from the literature.

References	As	Cd	Co	Cr	Cu	Fe	Mn	Se	Zn
**This study:**									

**All individuals**	**1.07 ± 0.74**	**0.06 ± 0.07**	**0.09 ± 0.10**	**8.43 ± 8.66**	**1.73 ± 0.99**	**66.74 ± 44.93**	**0.94 ± 0.79**	**1.54 ± 0.79**	**38.37 ± 29.80**
	**(0.34-2.97)**	**(0.01-0.25)**	**(0.01-0.37)**	**(1.57-34.27)**	**(0.72-4.26)**	**(18.99-210.55)**	**(0.28-3.73)**	**(1.02-3.83)**	**(9.20-150.58)**
**Lateral region**	**1.21 ± 0.75**	**0.08 ± 0.05**	**0.07 ± 0.08**	**5.31 ± 3.79**	**1.63 ± 1.07**	**54.82 ± 30.46**	**0.68 ± 0.37**	**1.49 ± 0.62**	**39.93 ± 32.58**
	**(0.43-2.97)**	**(ND-0.17)**	**(0.01-0.37)**	**(1.57-15.42)**	**(0.72-4.26)**	**(18.99-117.94)**	**(0.28-1.57)**	**(0.50-2.65)**	**(9.20-150.58)**
**Dorsal fin**	**0.45 ± 0.16**	**0.15 ± 0.09**	**0.18 ± 0.09**	**22.46 ± 11.08**	**2.19 ± 0.17**	**120.36 ± 64.74**	**2.13 ± 1.13**	**1.81 ± 1.46**	**31.38 ± 10.97**
	**(0.34-0.68)**	**(ND-0.25)**	**(0.09-0.27)**	**(11.79-34.27)**	**(2.00-2.41)**	**(71.26-210.55)**	**(1.14-3.73)**	**(0.62-3.83)**	**(19.35-41.66)**

**Other studies**									

[3]	-	-	-	-	3.1 ± 1.9(0.6–7.9)	176 ± 138(25.8–502)	2.2 ± 3.2(0.6–10)	-	210 ± 186(20.1–726)
									
[4]	1.6 ± 1.7 (1.0-1.9)	-	≤ 0.8	-	3.1 ± 0.2	14.2	3.1	2.1	-
					(2.9-3.2)	± 2.6	± 0.9 (2.8-4.9)	± 1.4 (1.1-3.3)	
						(12-16)			
[5]	-	≤0.09	-	≤0.7	1 ± 0.1	-	-	-	20 ± 1
					(0.9-1.1)				(19-21)
[6]	≈4.7 ± 6.8	<0.9	<0.9	<0.9	5.6 ± 8.2	-	–	5.2	9.2 ± 1.6
	(<0.8-12.6)				(0.9-15)			± 3.5	(7.67-10.82)
								(nd-7.67)	
[7]	-	-	-	-	-	-	-	8.96	-
								± 4.95	
								(2.09-21.29)	
									
[8] *	-	0.05*	-	-	-	-	-	-	-
[9] *	-	-	-	-	-	-	-	0.12	-
								± 0.04*	
								(0.089-0.174)	
[10] *	-	-	-	-	-	-	0.42 ± 1.29*	-	-
							(0.001-10.5)		
[11] *	-	0.07 ± 0.06*	-	-	0.36 ± 0.22*	40 ± 24*	0.42 ± 0.41*	-	10 ± 6.1*
		(0.01-0.19)			(0.14-0.95)	(18-106)	(0.11-1.6)		(3.4-28)

**Table 4:** Significant correlations between trace elements, lipids with body length. The Spearman correlation analysis was done only with data from samples of the lateral region. p<0.05 were considered as significant.

Significant correlations between trace elements and body length			
Trace elements	Body length	P-Value	

Cr	0.63	0.005	**
Co	0.65	0.003	**
As	0.62	0.006	**
Cd	0.74	0.000	***
Significant correlations between lipid species and body length			
			
Lipid species	Body length	P-Value	

CER.18.0.	-0.63	0.0053	**
CER.18.1.	-0.55	0.0169	*
CER.20.0.	-0.64	0.004	**
CER.20.1.	-0.63	0.0052	**
CER.22.0.	-0.50	0.0343	*
CER.22.1.	-0.70	0.0011	**
DCER.16.0.	-0.74	0.0005	***
DCER.18.0.	-0.63	0.0053	**
HCER.16.0.	-0.50	0.0365	*
HCER.22.0.	-0.55	0.0185	*
HCER.22.1.	-0.52	0.0287	*
CE(22:6)	-0.54	0.021	*
CE(14:0)	-0.59	0.0097	**
CE(16:0)	-0.51	0.0322	*
CE(16:1)	-0.56	0.0155	*
CE(20:5)	-0.50	0.036	*
TAG(46:2/FA16:1)	-0.48	0.0464	*
TAG(48:1/FA16:1)	-0.47	0.048	*
TAG(50:6/FA20:4)	-0.48	0.0417	*
DAG(14:0/16:1)	-0.52	0.0265	*
DAG(16:0/16:1)	-0.51	0.0319	*
DAG(16:1/16:1)	-0.47	0.0469	*
DAG(16:1/18:0)	-0.48	0.0453	*
LPE(16:1)	-0.66	0.0026	**
LPE(18:2)	-0.50	0.0334	*
PE(18:0/16:1)	-0.47	0.0481	*
PE(O-16:0/20:4)	0.49	0.0379	*
PE(P-16:0/18:0)	-0.49	0.0369	*

**Table 5:** Lipid significant differences related to biological features (Growth stage, stranding code and stomach content). The Wilcoxon rank-sum analysis was done only with data from samples of the lateral region. V represents the similarity between compared values. The further V-value is from zero, the smaller is p-value, meaning the more different are the medians. p<0.05 were considered as significant.

	V	P-value	-LOG10(p)	FDR
Growth stage				

PG(18:1/20:3)	59	0.018511	1.7326	0.99018
CER(20:0)	13	0.033011	1.4813	0.99018
CER(20:1)	14	0.043089	1.3656	0.99018
CER(22:1)	14	0.043089	1.3656	0.99018
STRANDING CODE				

PE(O-18:0/16:0)	47	0.015126	1.8203	0.94304
PE(O-18:0/20:4)	45	0.031933	1.4958	0.94304

STOMACH CONTENT				

PE(P-18:1/16:0)	75	0.00025138	3.5997	0.079437
PE(P-18:1/16:1)	71	0.0018854	2.7246	0.29789
PE(P-16:0/20:1)	67	0.011175	1.9518	0.47751
PE(P-18:0/16:1)	66	0.011375	1.944	0.47751
PE(P-18:0/18:2)	66	0.013971	1.8548	0.47751
PE(16:0/16:1)	65	0.015397	1.8126	0.47751
PE(P-18:1/22:6)	65	0.015397	1.8126	0.47751
PE(P-18:0/20:1)	65	0.01734	1.7609	0.47751
PG(18:2/18:2)	65	0.018294	1.7377	0.47751
LPE(16:1)	63	0.026772	1.5723	0.47751
PG(18:2/16:1)	63	0.028064	1.5518	0.47751
PG(18:0/22:5)	63	0.028896	1.5392	0.47751
PE(P-16:0/20:3)	63	0.029651	1.528	0.47751
PE(P-18:1/18:0)	61.5	0.040526	1.3923	0.47751
HCER(22:0)	61	0.044118	1.3554	0.47751
PE(P-16:0/20:4)	61	0.044118	1.3554	0.47751
PE(P-18:0/22:5)	61	0.044118	1.3554	0.47751
PE(P-18:0/22:6)	61	0.044118	1.3554	0.47751
PE(P-18:1/22:5)	61	0.044118	1.3554	0.47751
PG(18:1/18:2)	61	0.044118	1.3554	0.47751
PG(16:0/22:4)	61	0.045869	1.3385	0.47751

## 2 Experimental Design, Materials and Methods

Blubber samples from two areas (lateral and dorsal fin) were collected from bottlenose dolphin stranded along the Yucatan coast. All the collected samples were authorized by the Secretary of Environmental and Natural Resources of Mexico (SEMARNAT) with permits No. SGPA/DGVS/02797/16, SGPA/DGVS/00403/17, SGPA/DGVS/007603/18 and SGPA/DGVS/06727/19. The strandings were attended by the Program of Investigation and Conservation of Marine Mammals of Yucatan (PICMMY-UADY) from Universidad Autónoma de Yucatán. The samples were collected *in situ* from the lateral area, on the flank, below the dorsal fin of dolphins; except for dolphins Tt3, Tt6, Tt10 and Tt12, for which samples were collected from the lowest region of the dorsal fin. All the analysis was performed as is mentioned in [1]. Briefly, statistical processing was done using R software [2]. The mean, standard deviation and range were calculated for each element concentration. Correlation analysis between trace elements, lipids and body length was conducted using corr and Hmisc packages in the R software where p<0.05 was considered as significant. Metaboanalyst 4.0 online platform was used to determine differences between biological characteristics (stranding code, stomach content and growth stage) with the Wilcoxon rank-sum test; p<0.05 considered as significant. Both the correlation analysis and Wilcoxon rank-sum test were done only with samples from lateral region to avoid variability.

## Funding

This research is part of Cátedras CONACYT, a biotechnology of marine organisms project. This research was partially funded by the 10.13039/501100003141National Council of Science and Technology of Mexico (CONACyT) - Mexican Ministry of Energy - Hydrocarbon Fund, project 201,441. This is a contribution from the Gulf of Mexico Research Consortium (CIGoM). Ixchel M. Ruiz-Hernández thanks CONACyT for the doctoral fellowship. This study is part of the Small Projects Program 2019 supported from the Mexican Society of Marine Mammals (SOMEMMA). This project was made possible by U.S. 10.13039/100000002National Institutes of Health (NIH) Shared Instrumentation (Grant 1S10OD018141-01A1; to N.D.D.).

## CRediT authorship contribution statement

**Ixchel M. Ruiz-Hernández:** Conceptualization, Methodology, Software, Formal analysis, Investigation, Writing – original draft, Visualization, Funding acquisition. **Mohammad-Zaman Nouri:** Methodology, Formal analysis, Investigation. **Marianne Kozuch:** Methodology, Formal analysis, Investigation. **Nancy D. Denslow:** Methodology, Formal analysis, Investigation, Resources, Writing – review & editing, Supervision. **Raúl E. Díaz-Gamboa:** Resources, Writing – review & editing. **Rossanna Rodríguez-Canul:** Writing – review & editing, Supervision, Funding acquisition. **Reyna C. Collí-Dulá:** Conceptualization, Methodology, Formal analysis, Investigation, Resources, Writing – review & editing, Supervision, Project administration, Funding acquisition.

## Declaration of Competing Interest

The authors declare that they have no known competing financial interests or personal relationships that could have appeared to influence the work reported in this paper.

## Data Availability

MTBLS5772: Trace elements and lipidomic datasets of stranding samples in the blubber of Turpsios truncatus from the Yucatan Coast: Association with biological features (Original data) (MetaboLights).
